# Causal mechanisms of individual differences in hemispheric lateralization of the face perception network: A DCM-PEB approach

**DOI:** 10.1162/IMAG.a.1219

**Published:** 2026-04-24

**Authors:** Julia Elina Stocker, Peter Zeidman, Ina Thome, Kristin Marie Rusch, Jens Sommer, Olaf Steinsträter, Andreas Jansen

**Affiliations:** Department of Psychiatry and Psychotherapy, University of Marburg, Marburg, Germany; Wellcome Centre for Human Neuroimaging, Institute of Neurology, University College London, London, United Kingdom; Clinic of Neurology and Neurophysiology, Medical Center-, Faculty of Medicine, University of Freiburg, Freiburg, Germany; Center for Mind, Brain and Behavior (CMBB), Universities of Marburg, Gießen and Darmstadt, Germany; Core-Facility Brainimaging, Faculty of Medicine, University of Marburg, Marburg, Germany

**Keywords:** lateralization, face processing network, FFA, OFA, DCM, PEB

## Abstract

Functional lateralization is a fundamental organizational principle of the human brain, yet the neural mechanisms underlying inter-individual variability in hemispheric dominance remain poorly understood. In this study, we investigated the causal network dynamics contributing to hemispheric lateralization within the face perception system, focusing on the fusiform face area (FFA) and occipital face area (OFA). Using Dynamic Causal Modelling (DCM) combined with Parametric Empirical Bayes (PEB) in a large sample of 110 participants, we examined how individual differences in lateralization indices (LI) relate to effective connectivity in the bilateral core face network. Two complementary approaches were applied: a hypothesis-driven model comparison and additionally an exploratory model reduction. Both analyses consistently showed that lateralization in the FFA and OFA was explained by distinct network mechanisms. FFA lateralization was primarily driven by processes in the left hemisphere, reflected in a face-specific modulation of self-inhibition in the left OFA. Specifically, increased left-lateralization was associated with reduced self-inhibition (i.e., increased excitability) in the left OFA. In contrast, OFA lateralization depended on interhemispheric interactions involving both hemispheres, most prominently between the left and right FFAs. Notably, in both cases, lateralization arose from network-level interactions rather than changes within the regions themselves, highlighting the distributed nature of hemispheric specialization in face processing.

## 1 Introduction

The face perception network is bilaterally distributed across the brain, with key regions in the occipito-temporal cortex such as the occipital face area (OFA), the fusiform face area (FFA), and a face-sensitive region in the posterior superior temporal sulcus (pSTS) present in both hemispheres (for an overview on the neuroanatomical basis of the face perception network, see the reviews written by Duchaine & Yovel (2015) or Haxby et al. (2000)). The network has traditionally been considered right-hemisphere dominant. Lesion studies have shown that most patients with acquired prosopagnosia—an impairment in recognizing facial identity due to brain damage—typically have lesions in the right posterior hemisphere (for an overview, cf. Bukowski et al., 2013). In contrast, cases of prosopagnosia resulting from unilateral left-hemisphere lesions have been reported only rarely. The notion of right-hemispheric dominance has been further reinforced by extensive research spanning decades, including studies using behavioral experiments as well as brain stimulation techniques such as transcranial magnetic stimulation (TMS) and transcranial direct current stimulation (tDCS) (for an overview, see Duchaine & Yovel, 2015; Rossion & Lochy, 2022).

However, findings from functional magnetic resonance imaging (fMRI) studies present a more nuanced picture. While studies assessing hemispheric lateralization based on averaged activation across the entire core system or even larger brain regions (e.g., the entire temporal lobe) frequently report a clear right-hemispheric dominance (Badzakova-Trajkov et al., 2010), studies that analyze lateralization at the level of individual core system regions often reveal substantial inter-individual variability. In fact, up to 45% of participants in such studies do not exhibit right-hemispheric dominance for face perception (e.g., Davies-Thompson et al., 2016; De Winter et al., 2015; Johnstone et al., 2020).

To systematically investigate the presumed right-hemispheric dominance of the face perception network, we recently conducted a large-scale fMRI study (Thome et al., 2022). While our results showed that, on average, face-related activity was stronger in the right hemisphere, this asymmetry was only weakly pronounced compared for instance with the language network and exhibited considerable variability across individuals. Only approximately half of the participants displayed strong right-hemispheric dominance (i.e., lateralization index (LI) < -0.2), while the remaining half showed either bilateral or even left-hemispheric activation patterns. These findings suggest that the lateralization of face perception, as measured with fMRI, is far more complex than previously assumed, with substantial individual differences rather than a strictly right-dominant organization at the population level.

To fully understand the face perception network, it is essential to investigate the interactions between the involved regions. Up to now, there is still a lack of detailed network models that specifically describe how the two hemispheres interact. Since both hemispheres are typically engaged in face processing, efficient communication between them is necessary. This exchange is most likely mediated primarily by homotopic connections (e.g., left OFA—right OFA), rather than by the structurally weaker heterotopic connections (e.g., left OFA—right FFA) (Catani & Thiebaut de Schotten, 2008; Hofer & Frahm, 2006). The goal of the present study was to develop a network model that can specifically explain how inter-individual differences in the lateralization of fMRI activation patterns arise at a mechanistic level.

The interaction within a neural network can be investigated using neuroimaging techniques combined with suitable modeling methods. A particularly effective approach is Dynamic Causal Modelling (DCM) (Friston et al., 2003). DCM allows researchers to examine how experimental manipulations (e.g., task demands) influence neural activity within distinct brain regions and the directed interactions between them (“effective connectivity”). In its original implementation, DCM models neural dynamics using a bilinear differential equation (i.e., a low-order Taylor series approximation). For fMRI data, these predicted neural time series are translated into a blood oxygenation level-dependent (BOLD) signal time series via a hemodynamic model, allowing comparisons with the observed fMRI data. Together, the bilinear neural state equation and the hemodynamic model form a system, where experimental manipulations (input) are deterministically linked, either as direct input or as modulatory parameter on specific network connections, to the measured BOLD response in brain regions (output). This system can be inverted—typically using a Variational Bayes (VB) optimization scheme with Gaussian assumptions on the prior and posterior distributions (Zeidman et al., 2023)—to compare different neural models and estimate the posterior densities of model parameters (i.e., conditional mean and variance). In recent years, DCM has been used in various studies to investigate the face processing network (e.g., C. C. Chen et al., 2009; Fairhall & Ishai, 2007; Frässle, Krach, et al., 2016; Frässle, Paulus, Krach, & Jansen, 2016; Frässle, Paulus, Krach, Schweinberger, et al., 2016; He & Johnson, 2018; He et al., 2015; Kessler et al., 2020, 2021; Lee et al., 2022; Li et al., 2010; Lohse et al., 2016; Nagy et al., 2012; Nguyen et al., 2014). However, only a few studies have systematically included brain regions in both hemispheres in their models. To our knowledge, no study has yet attempted to uncover the neural mechanisms underlying inter-individual differences in hemispheric lateralization for face perception.

The standard DCM approach has recently been extended with a hierarchical model over parameters, called the Parametric Empirical Bayes (PEB) framework. PEB enables investigation into how network connectivity patterns vary across individuals in response to experimental manipulations. This relies on a hierarchical Bayesian approach. First, DCM is used to estimate a posterior probability distribution over model parameters (such as connectivity strengths) for each subject. These individual-level posteriors are then taken to the group level and explained using a General Linear Model (GLM), which has parameters encoding the effect of each hypothesized covariate (e.g., laterality index) on each neural connection. This method is particularly useful in neuroimaging studies with multiple subjects, as it allows for robust and statistically reliable conclusions about the neural mechanisms underlying group differences—for instance hemispheric lateralization (for an overview on PEB, see Zeidman et al., 2019). Since its inception, PEB has been applied to investigate interactions between brain regions, for example, in the motor system (Abidi et al., 2022; Bencivenga et al., 2021; Calzolari et al., 2023; Gandolla et al., 2021; Spedden et al., 2020), reward processing (Chan & Chou, 2022; Yc et al., 2023), social cognition (Q. Chen et al., 2024; Dietz et al., 2020; Esménio et al., 2020), language (Mizrachi et al., 2024), extinction learning (Paci et al., 2023), emotion processing (Rho et al., 2021), and response inhibition (Zhuang et al., 2023).

In summary, in this study we aim to develop network models that provide a mechanistic explanation for inter-individual differences in the hemispheric lateralization of face processing. To achieve this, we leverage a large-scale fMRI dataset of 110 participants who performed a classic face processing task (“face localizer”). This dataset has previously been used to descriptively characterize the lateralization of the face processing network (Thome et al., 2022). Using PEB and DCM, we identify the most plausible mechanisms underlying these lateralization differences. Specifically, we investigate three key factors that may independently contribute to altered hemispheric lateralization:

1. **Hemisphere:** Are lateralization differences driven by changes in the right hemisphere, the left hemisphere, or both? Increased right-hemispheric lateralization, for example, could result from heightened activity in the right hemisphere or reduced activity in the left.
2. **Regional specificity:** Do lateralization differences primarily emerge at the level of the OFA, the FFA, or at both?
3. **Network dynamics:** Are lateralization differences better explained by face-specific modulations of self-inhibition within a region or by altered connectivity between regions?

By systematically evaluating these factors, our study aims to move beyond descriptive findings and provide a causal understanding of the neural mechanisms shaping hemispheric lateralization in face perception.

## 2 Methods

### 2.1 Participants

Subjects were recruited in an fMRI study investigating the neural mechanisms underlying hemispheric lateralization (“Marburg 300 Study”). The dataset initially included 119 participants, but 9 were excluded due to poor MRI data quality. The final sample comprised 110 subjects (69 females, 41 males; mean age 24.5 years ± 3.6 years). Handedness was assessed using the Oldfield Handedness Inventory (1971, cut-off: ±30), revealing 85 right-handed, 23 left-handed, and 2 ambidextrous individuals.

The study was conducted in accordance with the Declaration of Helsinki and was approved by the local ethics committee of the medical faculty at the University of Marburg (file reference 160/13 version 2). All participants provided informed consent.

This dataset has previously been used to descriptively characterize the lateralization of the face processing network. Comprehensive details on participant recruitment and study design can be found in a prior publication from our group (Thome et al., 2022). Notably, in the previous study, ambidextrous participants (n = 2) were excluded, as handedness was treated as a categorical variable, resulting in a final sample size of 108 participants. In contrast, the present study (referred to as the “Marburg Sparta Study”) treats handedness as a continuous covariate. Therefore, these participants were not excluded.

### 2.2 Experimental procedure

Participants viewed either faces, houses, or scrambled images in a blocked-design fMRI experiment. Each block lasted 14.5 s. Nine blocks of each stimulus category were presented. In each block, 20 stimuli were presented. Each stimulus was presented for 300 ms and was followed by a fixation cross for 425 ms. Stimulus blocks were separated by baseline blocks of 14.5 s, where only a fixation cross was shown. Participants had to press a button when the same image appeared twice in a row (1-back task). The experiment lasted about 13 min. A more detailed description of the paradigm, including a graphical visualization of the stimuli, can be found elsewhere (Thome et al., 2022).

### 2.3 MR data acquisition

Data were collected with the 3 Tesla MR Scanner (TIM Trio, Siemens, Erlangen, Germany) at the Clinic for Psychiatry and Psychotherapy, University of Marburg. Functional images were acquired with a T2*-weighted gradient-echo echo-planar imaging sequence (TR = 1450 ms, TE = 25 ms, voxel size = 3 x 3 x 4 mm^3^, 30 slices, 4 mm thickness, flip angle = 90°, matrix size = 64 x 64 voxels, FoV = 192 x 192 mm^2^). All functional images were collected in descending slice order and parallel to the intercommissural plane (anterior to posterior commissure).

The anatomical images were acquired in sagittal orientation using a magnetization-prepared rapid gradient-echo (3D MP-RAGE) sequence (TR = 1900 ms, TE = 2.54 ms, voxel size = 1 x 1 x 1 mm^3^, 176 slices, 1 mm thickness, flip angle = 9°, matrix size = 384 x 384, FoV = 384 x 384).

### 2.4 MR data processing

#### 2.4.1 Preprocessing

Preprocessing of the MRI data was done using the software package SPM12 (Statistical Parametric Mapping, version v6015, Wellcome Trust Centre for Neuroimaging, London, UK; http://www.fil.ion.ucl.ac.uk). First, the initial four functional images were removed to accommodate for scanner instabilities. Second, to decrease motion artifacts, the functional images data were realigned to the mean image. Third, the high-resolution anatomical image was co-registered with the mean functional image, then segmented and normalized to the MNI template using the unified segmentation-normalization approach (Ashburner & Friston, 2005). All functional images were then normalized using the parameters obtained from the normalization of the anatomical image and resampled to a voxel size of 2 × 2 × 2 mm^3^. Last, the functional images were smoothed with an isotropic 6 mm full width at half maximum (FWHM) Gaussian kernel to enhance the signal-to-noise ratio of the data.

#### 2.4.2 Statistical analysis

A univariate general linear model (GLM) analysis with one regressor per condition (“faces”, “houses”, “scrambled images”) was performed to predict the blood oxygenation level-dependent (BOLD) signal. The three condition regressors were convolved with SPM’s canonical hemodynamic response function. Additional nuisance regressors, such as the six realignment parameters from the preprocessing steps and a high-pass filter with a cutoff at 1/128 Hz, were added to the model.

At the single subject level, face-sensitive brain activity was assessed using a conjunction analysis. This approach combined statistical maps for “faces > houses” and “faces > scrambled” images, whereby for each voxel the smallest t-value was chosen across both contrasts. Compared with a simple additive model, the conjunction map provides a more conservative estimate, as it includes only voxels that are significantly activated in both contrasts (for a detailed discussion, see Thome et al., 2022).

At the group level, individual contrast images (“faces > houses”, “faces > scrambled” images) were included in a random-effects analysis (one-way ANOVA, two levels). For each level, we computed a separate contrast using the weights “1 0” and “0 1”, respectively. Face-sensitive brain activity was then identified through a conjunction analysis of these contrasts.

### 2.5 Calculation of hemispheric lateralization

Hemispheric dominance was assessed using a lateralization index (LI), computed separately for the OFA (LI_OFA_) and the FFA (LI_FFA_). The LI provides a measure of whether activation within a given region is predominantly left-hemispheric, right-hemispheric, or bilaterally distributed. It is calculated as the difference between activity in the left (A_L_) and right (A_R_) region-of-interest (ROI), normalized by their sum:

$$LI=\;\frac{A_{L}-A_{R}}{A_{L}+A_{R}},$$ (1)

where A_L_ and A_R_ denote the fMRI-measured activity within the respective ROIs (Jansen et al., 2006). The LI ranges from -1 to 1, with positive values indicating left-hemispheric dominance, negative values indicating right-hemispheric dominance, and values close to zero reflecting bilateral activation. Bilateral activity is conventionally defined using a threshold of ±0.2 (Jansen et al., 2006). This approach provides a straightforward and interpretable index for comparing hemispheric dominance across participants and regions.

#### 2.5.1 Choice of activity measure

In lateralization research, several approaches have been established to quantify the strength of brain activity (i.e., A_L_ and A_R_). The most widely used measures are either based on the magnitude of the fMRI signal change (e.g., weighted β-values or t-values) or on the extent of the activated brain region (i.e., number of activated voxels) (for a detailed overview, see Jansen et al., 2006). In the present study, we used t-values as the measure of activity.

All LIs were computed using the bootstrap procedure (Wilke & Schmithorst, 2006) introduced by Wilke and Schmithorst and implemented in the LI toolbox (version 1.3, Wilke & Lidzba, 2007) for SPM12 (MATLAB version 2017a). The procedure consisted of several steps:

1. **Thresholding and ROI masking:** Individual conjunction t-maps were thresholded and masked with custom ROI masks for the left and right OFA and left and right FFA (see below for ROI creation).
2. **Bootstrapping:** From all surviving voxels, 100 bootstrapped samples were drawn from each hemisphere (resampling ratio k = 0.25, with replacement), and all possible LI combinations (10,000) were calculated and plotted as a histogram.
3. **Trimmed mean calculation:** A “trimmed mean” LI was computed from the central 50% of bootstrapped LI values. This procedure was repeated for 20 regularly spaced threshold steps. Of note, we chose to use the trimmed mean for the LI calculation to obtain a robust estimate of hemispheric dominance. By removing a small proportion of the highest and lowest voxel values within each ROI, this approach minimizes the influence of extreme values that can arise from noise or artifacts, while preserving the central tendency of the activation. This ensures that the resulting LI values are more stable and comparable across subjects and sessions.
4. **Weighted overall LI:** Finally, a weighted overall mean LI was calculated using the t-threshold as a weighting factor, so that statistically more conservative thresholds received stronger weights.

This approach ensures that the LI reflects robust hemispheric differences while accounting for both voxel-wise variability and thresholding effects. A more detailed description of the bootstrapping procedure and toolbox implementation can be found in Wilke and Schmithorst (2006) and Wilke and Lidzba (2007), respectively.

#### 2.5.2 Definition of regions of interest

Ideally, ROI masks should capture the relevant brain activation (sensitivity) while excluding unrelated activations (specificity). ROIs can be defined either anatomically (based on structural landmarks) or functionally (based on activation patterns). Since OFA and FFA are closely adjacent, we opted for functionally defined ROIs.

The precise localization of face perception areas varies substantially across individuals. Consequently, we did not apply a single ROI mask for all subjects, but instead created subject-specific masks using the following procedure:

1. **Anatomical restriction:** For each ROI, we first generated symmetrical, box-shaped masks using the WFU PickAtlas (v. 3.0.5; Maldjian et al., 2003, 2004). The masks’ center coordinates and spatial extent were based on typical locations of the OFA and FFA reported in previous fMRI face-localizer studies (Berman et al., 2010; Fox et al., 2009; Pitcher et al., 2011; Rossion et al., 2012). These relatively large masks served as anatomical constraints: the subject-specific ROI centers had to lie within these boxes (see below). Coordinates are summarized in an OSF repository (https://osf.io/s8gwd/).
2. **Group-level peak identification:** Brain activation patterns were assessed at the group level. For each ROI, peak voxels were identified in the group-level conjunction contrast at p < 0.05, FWE corrected (Supplementary Table T1).
3. **Subject-specific peak selection:** For each subject, all local maxima of the individual conjunction t-map were determined that (i) fell within the anatomical mask, (ii) had a t-value ≥ 3.1 (p < 0.001, uncorrected), and (iii) were at least as high as the t-value at the group maximum coordinate.
4. **Final coordinate selection:** The local maximum closest to the group maximum was chosen. If no local maximum met these criteria, the group maximum coordinate was used for that subject.
5. **ROI mask creation:** Custom spherical masks with a 10 mm radius were then centered on the individually determined coordinates.
  All steps were implemented using custom MATLAB scripts; further details and a graphical overview of individual ROI centers are reported in a previous publication from our group (Thome et al., 2022).

### 2.6 Dynamic causal modelling analysis

We applied DCM12.5 (7479) in SPM12 (v7738) to investigate the effective connectivity in the face perception network. In the following, we describe the determination of ROIs for the DCM analysis as well as the extraction of time series, the definition of model space, and the model estimation procedure.

#### 2.6.1 Determination of ROIs and extraction of time series

**Fig. 1.**
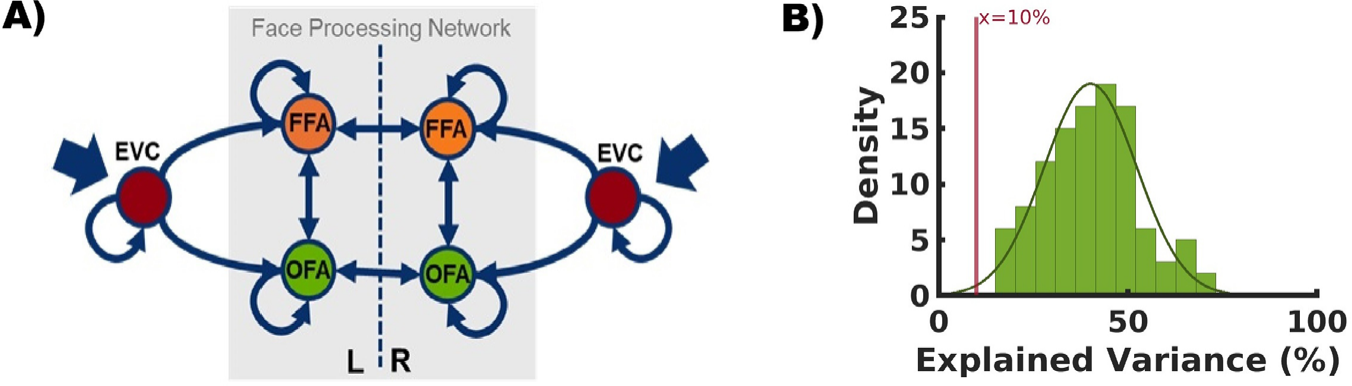
Model space and model accuracy. (A) The DCM network structure, including key face processing regions (FFA, OFA) as well as the early visual cortex (EVC) in both hemispheres. Directional connectivity (A-matrix) is represented by thin blue arrows connecting these regions. The driving input from visual stimulation (i.e., by faces, houses, and scrambled images) enters the left and right EVC, as indicated by thick blue arrows (C-matrix). Notably, modulatory input via face-specific stimulation is applied to all connections except for the self-connections of the EVCs, while modulatory influences from visual stimulation affect all six self-connections. (B) Density distribution of estimated model accuracies for all participants (n = 110). The horizontal axis is the percent explained variance of the fMRI time series. The cutoff value at 10% is highlighted in red. No participant was below this threshold.

The neural models for the DCM analysis included six regions: the left OFA (lOFA), right OFA (rOFA), left FFA (lFFA), right FFA (rFFA), and the left early visual cortex (lEVC) and the right early visual cortex (rEVC) as input regions (Fig. 1A). Since the exact localization of these regions varies between individuals, we determined them individually for each subject.

For the OFAs and FFAs, we used the same center coordinates as those applied for the LI calculation (see Section 2.5). The EVCs were localized using a contrast comparing visual input (faces, houses, scrambled images) against the implicit, non-modeled baseline. We identified individual maxima within anatomical masks encompassing Brodmann area 17 (left and right), generated with the WFU-Pickatlas (2D dilation: 1 voxel in each direction; Maldjian et al., 2003, 2004).

For each ROI, we extracted the BOLD signal time series as the first eigenvariate from all voxels within a 6 mm radius around the subject-specific ROI center coordinates. Only voxels exceeding a statistical threshold of p < 0.01 (uncorrected) were included in the calculation. For the OFAs and FFAs, we applied the conjunction contrast (see Section 2.4), while for the EVCs we used the baseline contrast. The time series were mean centered, and movement-related variance was removed using an effects-of-interest F-contrast.

Of note, laterality measures and DCM parameter estimation were not circularly related. Specifically, LIs were not used to define ROIs, to select data, or to constrain the DCM model structure or model space. Instead, LIs were included exclusively as between-subject covariates in the PEB design matrix, with the aim of explaining inter-individual variability in effective connectivity parameters after subject-level DCM estimation. Importantly, DCM parameters were estimated independently for each participant based solely on their time series data. The subsequent PEB analysis then modeled how these independently estimated parameters covary with laterality across participants. Thus, laterality does not bias the estimation of connectivity parameters, but rather serves as an explanatory variable for between-subject differences.

#### 2.6.2 Definition of model space

The neural network structure is described in Figure 1A. The model included six regions: the left and right EVC, the left and right OFA, and the left and right FFA. The OFAs and FFAs were included in the network model as key regions of the core system of face perception. The EVCs were included as visual input regions to the network. We used the standard fMRI neural model (bilinear, single state, and deterministic) for DCM. Notably, we specified only a single model at the individual subject level. Unlike the standard DCM approach, PEB-DCM does not involve comparing multiple models at the single-subject level. Instead, Bayesian model comparison is introduced at the group level to assess between-subject effects. This approach assumes that while all participants share the same fundamental network architecture (i.e., same model space), they may differ in the strength of connections within that model (Zeidman, Jafarian, Seghier, et al., 2019).

The intrinsic structure (A-matrix) was defined by bidirectional connections between OFA and FFA in each hemisphere, bidirectional interhemispheric connections between both OFAs and between both FFAs, and unidirectional forward connections from EVC to ipsilateral OFA and to ipsilateral FFA. Additionally, as implemented by default in DCM, all regions included self-connections. Of note, our model does not assume a hierarchy between OFA and FFA (Rossion & Lochy, 2022).

To model external manipulations (i.e., external input via the C-matrix or modulatory input via the B-matrix), we created a new SPM design matrix with two regressors. The first regressor represented “visual stimulation” and included the conditions faces, houses, and scrambled images (visual stimulation regressor). The second regressor included only the faces condition, thereby capturing face-specific influences on the network structure (face regressor). The driving input to the system was modeled by the visual stimulation regressor, targeting both EVC regions. Face-specific modulations were applied to all possible connections, including the self-connections of the OFAs and the FFAs, but excluding the self-connections of the EVC regions. To account for potential modulatory influences of visual input, we additionally applied the visual stimulation regressor to all six self-connections.

The same neural model has been used in a similar manner in several previous studies conducted by our group. A comprehensive discussion on the selection of regions, their connections, as well as the driving and modulatory input can be found elsewhere (Stocker et al., 2025).

The connectivity changes between regions are characterized as the influence of one region on the average activity of the other (in unit Hz). For between-region connections, positive connectivity parameters indicate an excitatory connection between the regions, whereas negative connections are inhibitory. An exception are self-connections that are always negative, downregulating the region’s neural response by a factor of -0.5 exp(x), where -0.5 Hz is the default level of self-inhibition and x is the estimated connectivity parameter. Negative self-connections thus stand for the natural decay or damping of neural activity.

Of note, in our model design we decided to drive both the modulatory self-connections of the B matrix and the input on EVC by the visual regressor. This way our visual input serves two roles and might appear difficult to distinguish, favoring potential collinearities between the posterior parameters of B and C matrices. However, as shown in a post hoc analysis, we only observed weak average correlations between the visual input and its modulation of self-connections (r = 0.02, SD = 0.13). We thus can justify choosing to include visual modulatory self-connections, especially as they serve to account for local changes that are merely driven by visual effects (faces, houses, scrambled images) not by faces alone. This way, we prevent biasing our model toward face-driven explanations and allow the model to separate generic visual processing from face-specific modulations more effectively. We confirmed the robustness of this type of modeling also by rerunning the analysis with visual modulatory effects only on the EVC self-connections, excluding the OFA and FFA self-connections. Here we retain similar results to our original findings. These results of this analysis are given in Supplementary Table T6.

#### 2.6.3 DCM model estimation

We then estimated (i.e., inverted) each subject’s DCM using a variational Laplace estimation scheme. This process involves determining the posterior density over parameters that maximizes a score for the quality of the model, called the negative variational free energy (hereafter, the free energy). The free energy serves as an approximation of the log probability of having observed the data, given the model (log model evidence). This Bayesian model inversion, therefore, returns two outputs: an estimate of the log model evidence and a multivariate normal probability density over the model parameters. These estimated parameters serve as input for the subsequent PEB analysis (see Section 2.7).

In Figure 1B, we present the distribution of explained variance across participants. As the explained variance exceeds the default cutoff value of 10%, we deemed the estimated connectivity parameters sufficiently useful to proceed with further analyses.

### 2.7 PEB analysis

After retrieving the expected values of the connectivity parameters along with their probability distribution for each DCM, we ran a PEB analysis. This approach enabled us to construct a random-effects GLM to explain the connectivity parameters by capturing both commonalities and differences across participants. The GLM is defined by the following equation:

θ=Xβ+ε,

where θ represents the estimated connectivity parameters for all subjects, X is the design matrix including chosen regressors, *β* is the final group-level parameters, and *ε* is the unexplained variance between participants. The goal of PEB is to estimate the *β* values. To do so, it estimates the joint probability of the data, the subject-specific parameters θ, and the group-level parameters *β*.

To enhance statistical efficiency, only DCM parameters relevant to the tested hypotheses are typically carried forward to the group level. For the present analysis, we included all 22 modulatory parameters. As a reminder, the visual stimulation regressor was applied to all six self-inhibitory connections. The face regressor was applied to the four self-inhibitory connections of both OFAs and both FFAs, to the four interhemispheric connections between the OFAs and the FFAs, to the four intrahemispheric forward connections from the EVCs to the OFAs and FFAs, and to the four intrahemispheric connections between the OFAs and FFAs.

In the following, we describe specification of the PEB design matrix X, the PEB model estimation, and the final inference.

#### 2.7.1 PEB: Design matrix specification

The second-level design matrix X defines the hypotheses about between-subject variability. It consists of two parts: between-subject effects and within-subjects effects.

The between-subject design matrix defines which between-subject variables have a potential influence on the DCM parameters. We chose one regressor representing commonalities across participants and five regressors capturing individual differences. The commonality regressor reflects the mean connectivity values for all parameters and was implemented as a column of ones. The five additional covariate regressors included the lateralization indices for the FFA (LI_FFA_) and the OFA (LI_OFA_), the handedness score, and participant’s age and gender. The LIs were our primary variables of interest, while the remaining regressors were included to control for potential confounds. All covariates were mean centered. As a result, the commonality regressor represents the mean connectivity change across participants, whereas the additional regressors capture additions or subtractions from this mean.

The within-subject design matrix defines which of the 22 selected DCM parameters can receive between-subject effects. In the present study, we configured the matrix so that all six regressors (i.e., the commonality regressor and the five between-subject covariates LI_FFA_, LI_OFA_, handedness, age, and gender) could influence all connectivity parameters. Previously, handedness and age have been shown to modulate both the interhemispheric functional connectivity between homologous brain regions as well as the lateralization of intrahemispheric functional integration patterns (Jin et al., 2024).

#### 2.7.2 PEB: Model estimation and inference

After the specification of the PEB design matrix, we inverted the model. This gave us the estimated group-level parameters ß as well as the group-level free energy for the full model.

In the next step, we created different reduced PEB models by switching on and off different parameters. A parameter was switched off by fixing its value to zero (by setting its prior variance to zero). We then compared these models based on their free energy. This allowed us to identify the PEB models with the highest free energy and thus determine the optimal explanation for the dataset as a whole. The evidence and the parameters of reduced models (i.e., models with certain parameters switched off) were derived from the full model (the model in which all parameters were switched on) using a Bayesian Model Reduction (BMR) procedure (Zeidman, Jafarian, Seghier, et al., 2019). Subsequently, we thresholded our parameters to only report those with non-trivial posterior probability (Zeidman, Jafarian, Seghier, et al., 2019).

For inference, we pursued two complementary approaches. In the first analysis, we applied a hypothesis-driven approach by grouping models according to three experimental factors “hemisphere” (left, right), “region” (OFA, FFA), and “type of connections” (input connection, self-connections, intra- and interhemispheric connection). In the second analysis, we applied a hypothesis-free approach by using BMR to perform an automatic search over reduced models, removing parameters that did not contribute to the model evidence.

In these analyses, we focused on the influence of three regressors: the commonality regressor, which describes the modulation across participants, and the influence of the two regressors that describe the lateralization of the network (LI_OFA_ and LI_FFA_).

#### 2.7.3 Analysis 1 (Hypothesis-driven): Investigation of a reduced model space

For the hypothesis-driven approach, we defined a set of a priori chosen models to identify the best explanations for the commonalities and differences across subjects. The models varied in which connections were modulated by the face regressor. Notably, in all models, the visual stimulation regressor modulated the self-connections of all regions.

**Fig. 2.**
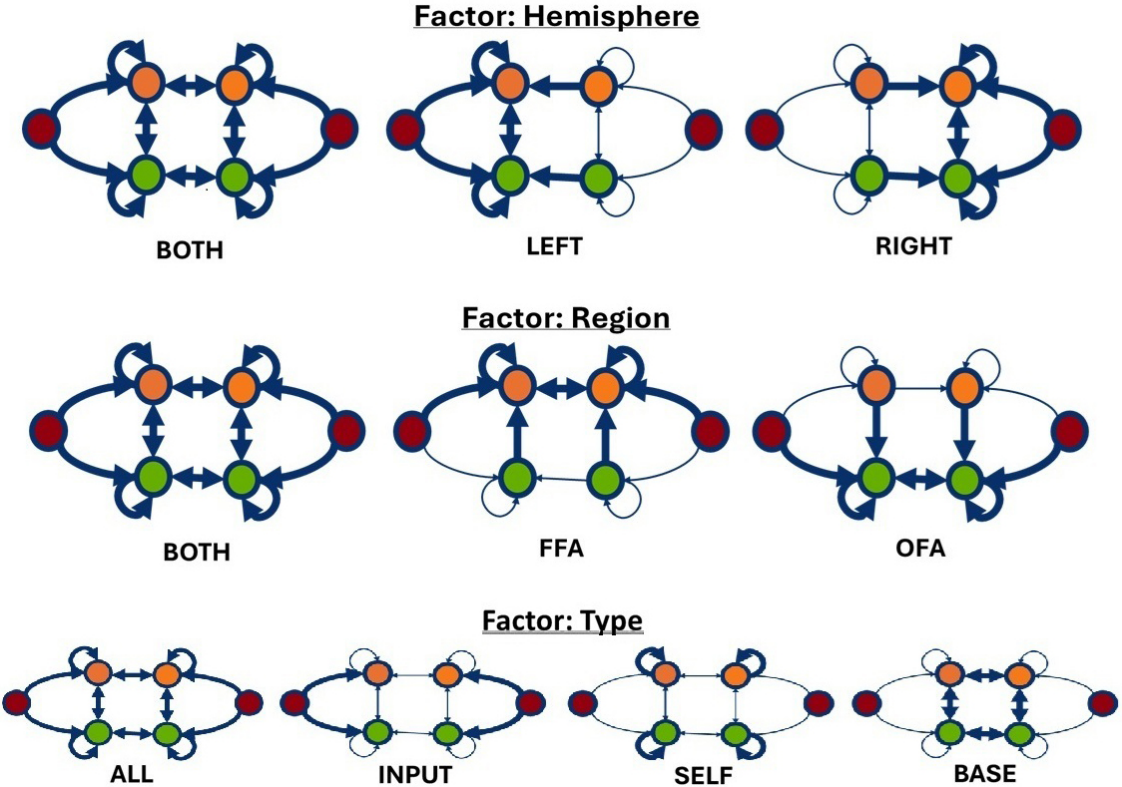
Hypothesis-driven model space. Red circles are left and right EVC, green circles are left and right OFA, orange circles are left and right FFA. Bold arrows indicate connections that were allowed to be modulated by the face regressor. The models were constructed using a factorial model space with three factors: “hemisphere,” “region,” and “type” (type of connections). Different PEB models were varied according to three factors (hemisphere, region, type of connections), giving 3 x 3 x 4 = 36 models. Additionally, a null model was defined in which no modulations by the face regressor was allowed (not shown).

The models were constructed using a factorial model space with three factors: “hemisphere,” “region,” and “type of connections.” The “hemisphere” factor had three levels: (1) left hemisphere (LEFT), (2) right hemisphere (RIGHT), and (3) both hemispheres (BOTH). The “region” factor also had three levels: (1) OFA, (2) FFA, and (3) both OFA and FFA. The “type of connection” factor had four levels: (1) input connection, where the forward connections from the EVCs to the OFAs and FFAs were modulated (INPUT); (2) self-connections, where the self-connections of the OFAs and FFAs were modulated (SELF); (3) intra- and interhemispheric connections, where the connections between the OFAs and FFAs were modulated (BASE); and (4) all connections (see Fig. 2 for a graphical illustration). For example, a model defined by the factor levels “hemisphere: left,” “region: OFA and FFA,” and “type of connections: self-connections” would exhibit face-specific modulation of the self-inhibitions of the lOFA and lFFA.

In total, this resulted in 3 × 3 × 4 = 36 models, defined by the different factor levels. Additionally, we created, as control model, a model without any modulations by the face regressor (“null model”). Using BMR, we then calculated the model evidence and the parameters of all reduced models from the full model.

Due to the large number of models, we did not expect a single model to emerge as the clear winner (i.e., with a posterior probability (Pp) of >95%). We, therefore, did not primarily analyze the evidence for each individual model separately, but rather we grouped the resulting models into different families based on the three factors and analyzed the pooled evidence. This allowed us to determine whether face stimuli predominantly modulated the left or right hemisphere, primarily influenced the OFA or FFA, and which types of connectivity parameters were modulated.

To summarize the parameters across all models, we used Bayesian Model Averaging (BMA) to compute the average of the parameters from different models, weighted by the models’ posterior probabilities (Penny et al., 2004).

#### 2.7.4 Analysis 2 (Hypothesis-free): Exploration of reduced models

In a complementary, exploratory analysis, we began with the full model and systematically eliminated model parameters that did not enhance model evidence. All reduced models were considered equally probable a priori.

The full model comprised 132 parameters, resulting from 6 between-subject regressors applied to 22 DCM connectivity parameters. Our objective was to identify the optimal model by switching off certain parameters.

Given the vast number of possible models (2^132^), calculating model evidence for each was unfeasible. Consequently, we employed an automatic search procedure known as Bayesian model reduction (Zeidman, Jafarian, Seghier, et al., 2019). BMA was then performed over the 256 models from the final iteration.

## 3 Results

In this study, our goal was to examine how the inter-individual differences, captured by subject-specific covariates, relate to the connectivity of the face processing network. We began by fitting a DCM to each subject’s fMRI time series data (see Supplementary Table T2 and Fig. S2 for the mean DCM parameters across all participants using Bayesian Parameter Averaging (BPA)). These models included parameters quantifying the modulatory effect of faces on all connections. We took these face modulation parameters to the group level, and modeled them in a regression model using the PEB framework (see Supplementary Table T3 for the non-reduced PEB parameters). The regression model included covariates for the average effect of each face parameter across subjects (hereafter, “commonalties”), and the effects of two laterality indices LI_FFA_ and LI_OFA_ on each face parameter.

Below, we present the results of comparing the evidence for the full regression model described above, against reduced models where particular effects were switched off. We begin with a hypothesis-driven analysis (Section 3.1), followed by an exploratory analysis (Section 3.2).

### 3.1 Analysis 1 (Hypothesis-driven): Investigation of a reduced model space

#### 3.1.1 Model comparison

For the hypothesis-driven approach, we had defined a set of 37 candidate regression models based on the factorial model space incorporating the factors “hemisphere,” “region,” and “type of connections.” We identified the models that best explained the commonalities across participants, and the inter-individual differences associated with two measures of laterality: LI_FFA_ and LI_OFA_. Notably, the two laterality measures exhibited only a weak correlation across participants (r = 0.08; see Supplementary Fig. S1). If they would have been highly correlated, this may have compromised our efficiency to detect unique effects associated with each.

We would like to note that, in the standard implementation of PEB in SPM, only the joint model probability across all 37 models—as expressed in terms of the common effects (first column of the PEB design matrix) and the first contrast of group differences (second column)—would have been computed. However, since our primary interest lay in the lateralization indices LI_FFA_ and LI_OFA_, we adapted the spm_dcm_peb_bmc subroutine to directly assess the contribution of the three first regressors.

We begin by presenting the overall winning model across all combinations of the three regressors. We then take a closer look at the best-fitting model for each covariate (Commonalities, LI_FFA_, LI_OFA_). To better understand the role of each experimental factor, we then grouped the models into families based on “Hemisphere,” “Region,” and “Type (of connections),” and identified the most likely model within each group by comparing the cumulative model evidence.

In the first step, we compared the evidence for a total of 37^3^ = 50,653 models. The best model combination (model 1, model 15, and model 29) had a joint posterior probability of 0.32. This relatively low probability was not unexpected, given the extensive size of the model space.

**Fig. 3.**
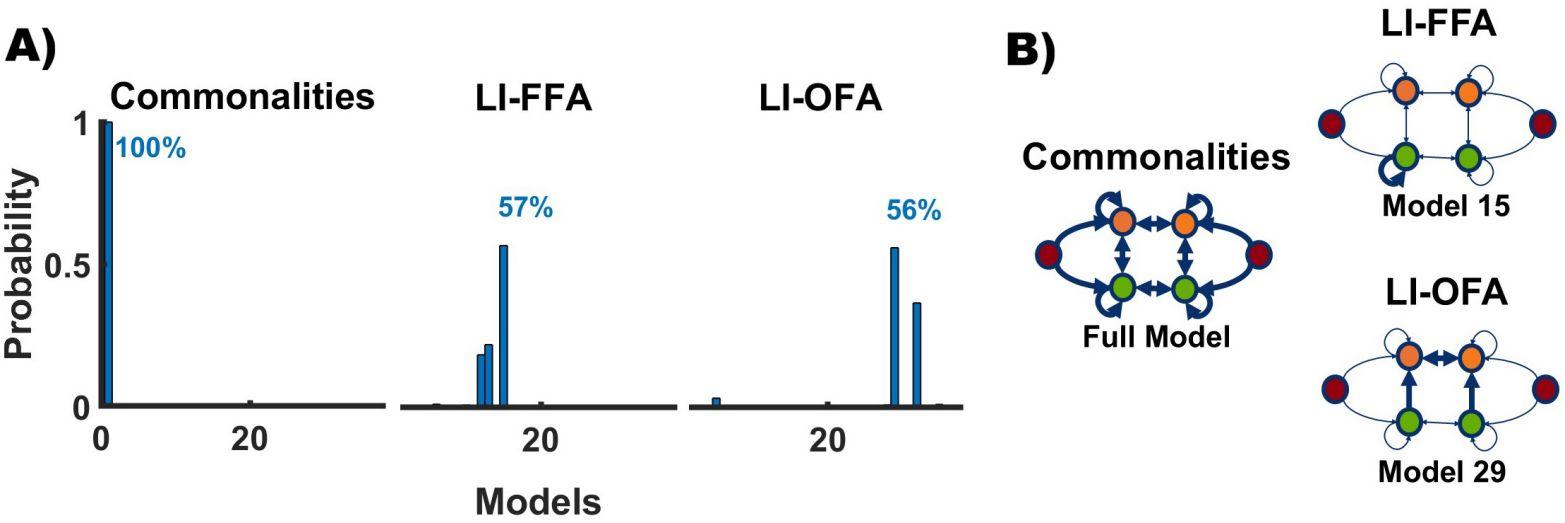
Posterior estimates and winning models. (A) Posterior probabilities for commonalities across participants (left), interindividual LI_FFA_ differences (middle), and interindividual LI_OFA_ differences (right). (B) The corresponding winning models are illustrated in right-hand panel. The full model (model 1) was the best explanation for commonalities across subjects. Model 15 was the best explanation for LI_FFA_ differences (Pp = 0.57). Model 29 was most probable for LI_OFA_ differences (Pp = 0.56).

In the second step, we calculated the posterior probabilities for each model separately for the commonalities across subjects (Fig. 3A left), the inter-individual LI_FFA_ differences (Fig. 3A middle), and the inter-individual LI_OFA_ differences (Fig. 3A right). Here, we reduced the complexity of the model space by marginalizing over the models associated with the other two external variables in each case. The full model (model 1) was the best explanation for commonalities across subjects (Pp > 0.999). Model 15 (Fig. 3B) was the best explanation for LI_FFA_ differences (Pp = 0.57). This model was characterized by face-specific modulations of the self-connection of the left OFA. For the LI_OFA_ differences, model 29 showed the highest posterior probability, with a value of 0.56. This model was characterized by face-specific modulations of the interhemispheric connections between FFAs and the intrahemispheric forward connections from lOFA to lFFA and from rOFA to rFFA.

In the third step, we grouped the candidate models into families and assessed which covariate was best explained by which family. For the factor “Hemisphere,” we sorted the models into the families “LEFT,” “RIGHT,” “BOTH,” and “NONE.” The best explanation for commonalities across participants was found for the family “BOTH” (Pp = 1.00). In contrast, LI differences were best explained for the FFA by the family “LEFT” (Pp(LI_FFA_) = 0.78) and for the OFA by the family “BOTH” (Pp(LI_OFA_) = 0.54). The factor “Region” was split into the families “FFA,” “OFA,” “BOTH,” and “NONE.” Here we found that commonalities were best explained by the family “OFA” (Pp(Commonalities) = 1.00), while the LI differences were again best explained for the FFA by the family “OFA” (Pp(LI_FFA_) = 0.75) and for the OFA by the family “FFA” (Pp(LI_OFA_) = 0.93). Finally, the factor “Type” sorted families into “INPUT,” “SELF,” “BASE,” or “ALL” connections. The null model was included as well. Here we found that the commonalities could also be best explained by the full model “ALL” (Pp(Commonalities) = 1.00). The lateralization differences of the FFA were best explained by the “SELF” model family (Pp(LI_FFA_) = 0.97). The LI_OFA_ differences could be explained by the “BASE” model family with a posterior probability of 92%.

In summary, all analyses converged on the same general pattern: inter-individual differences in LI_FFA_ were most consistently associated with face-specific modulation of self-inhibition in the left OFA. In contrast, interindividual differences in LI_OFA_ were best explained by face-specific modulation of inter-regional connectivity, specifically the interhemispheric connections between the FFAs and the intrahemispheric forward connections from OFAs and FFAs. Together, these results suggest different network mechanisms for the two lateralization covariates.

#### 3.1.2 Model averaging

**Fig. 4.**
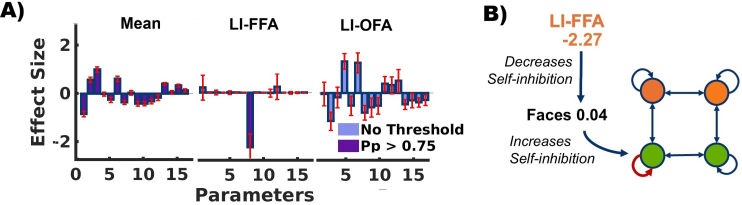
Bayesian Model Average (BMA) of the neural model parameters over all models. (A) The effect sizes of the parameters of primary interest (commonalities, LI_FFA_ and LI_OFA_). Effect sizes above the free energy threshold (Pp > 0.75) are presented in purple, others are shown in light blue. Parameters reduced to zero are symbolized by a line. Red interval lines show the standard deviations from mean. For a table with all parameter values, see Supplementary Table T4. (B) Influence of the FFA lateralization on the face processing network. Green circles at the bottom represent left and right OFA, orange circles at the top are left and right FFA. As shown by the commonality regressor (average connectivity over all subjects), face processing increases self-inhibition on left OFA. Thus, there is a decrease in activation when faces are presented. A higher (i.e., more positive) FFA lateralization index (LI) increases the face processing influence on the lOFA. Left lateralization of FFA activity is thus associated with a reduction in self-inhibition of the lOFA.

Apart from model comparison or selecting a winning model, we were interested in the actual connectivity parameters of our model. Thus, we computed the weighted average of all 22 neural model parameters across the 37 candidate models. This was done using Bayesian Model Averaging (BMA) which incorporates all six covariates within the PEB model, all models within the model space, and their posterior probabilities as weights. This procedure yielded a total of 132 group-level parameter estimates (22 x 6). The averaged parameters for the covariates of primary interest (i.e., the commonality parameter and the two lateral indices: LI_OFA_ and LI_FFA_) are shown in Figure 4A. To aid interpretation, we applied a posterior probability threshold of 0.75 to the effect sizes of connection strength, thereby highlighting those parameters with the strongest evidence of being non-zero. Importantly, none of the additional covariates (age, handedness, gender) were associated with any parameter at this threshold. A full list of all connectivity parameters is given in Supplementary Table T4 in the Supplementary Material.

Figure 4B exemplarily illustrates the neural network underlying interindividual differences in LI_FFA_. Our results provide a simple mechanistic interpretation of these differences. At the descriptive level, increased lateralization (i.e., more positive lateralization indices) reflects relatively stronger activation of the left compared with the right FFA. At the mechanistic level, increased lateralization is associated with a face-specific reduction in self-inhibition (LI_FFA_(lOFA_self_) = -2.27). Reduced self-inhibition corresponds to increased neural activity, thus providing a direct link between the observed modulation pattern and enhanced left-hemispheric FFA activation.

### 3.2 Analysis 2 (Hypothesis-free): Exploration of reduced models

**Fig. 5.**
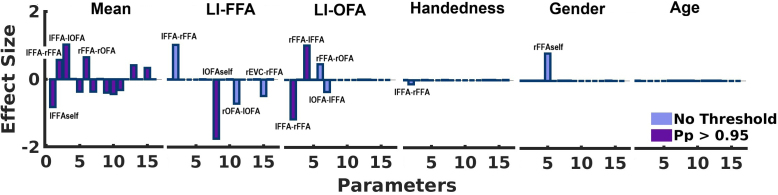
Group-level face-modulated connectivity. Posterior parameters retrieved from Bayesian Model Reduction (BMR). The effect sizes of the parameters are shown for all covariates (mean, LI_FFA_, LI_OFA_, Handedness, Gender, and Age). Effect sizes above the free energy (Pp > 0.75) threshold are presented in purple, others are shown in light blue. Parameters reduced to zero are symbolized by a line.

As a complementary and exploratory approach, we began with the full model and iteratively removed all model parameters that did not improve model evidence. The full model comprised 132 parameters (6 between-subject effects applied to 22 DCM parameters). A BMA was then computed across the 256 models retained in the final iteration of the automatic search procedure. The results are shown in Figure 5. As before, we report effect sizes for all parameters, but highlight those with a posterior probability exceeding 0.75, indicating strong evidence of a non-zero effect.

The estimated parameters were largely consistent with those identified in the hypothesis-driven analysis. Specifically, inter-individual differences in the lateralization of FFA activity (LI_FFA_) were again associated with a face-specific modulation of self-inhibition in the left OFA (LI_FFA_(lOFA_self_) = -1.75). Commonality parameters of the OFA self-inhibition remained similar (Commonalities(lOFA_self_) = 0.00). Inter-individual variation in LI_OFA_ was associated with face-specific modulation of the interhemispheric connections between FFAs (LI_OFA_(lFFA**→**rFFA = -1.16; LI_OFA_(rFFA**→**lFFA = 1.01). Thus higher lateralization values (more left lateralized) of the OFA lead to decreased modulation from left to right FFA and vice versa. Other covariates, those of no interest, did not show any significant associations with network dynamics. For a more detailed description of all parameter values, see Supplementary Table T5 in the Supplementary Material.

In summary, this exploratory analysis supports our previous findings by demonstrating that individual differences in LI_FFA_ can be explained by changes in the self-inhibition of left OFA. Moreover, it also highlights associations between LI_OFA_ and interhemispheric connectivity parameters describing interaction between both FFAs.

## 4 Discussion

Dynamic Causal Modelling (DCM) is a computational framework designed to infer and quantify directed (causal) interactions among brain regions, based on neuroimaging data such as fMRI. It models how one neural system exerts influence over another, thereby allowing us to understand the underlying neural mechanisms of cognitive processes. Parametric Empirical Bayes (PEB) extends DCM to the group level through a hierarchical Bayesian approach. It allows for the analysis of inter-individual variability in effective connectivity by treating the subject-specific DCM parameters as inputs to a Bayesian General Linear Model. This framework estimates group-level effects, accounting for both fixed effects (shared across participants) and random effects (subject-specific deviations),

Using PEB-DCM, the present study investigated how inter-individual differences in hemispheric lateralization, specifically in the FFA (i.e., LI_FFA_) and the OFA (i.e., LI_OFA_), are reflected in the neural network underlying face processing. We conducted two complementary analyses. In the first (hypothesis-driven) analysis, we defined a model space consisting of 37 candidate models, derived from a factorial design incorporating the factors “hemisphere,” “region,” and “type of connections.” In the second (exploratory) analysis, we employed a full model and applied a Bayesian model reduction procedure, iteratively pruning parameters that did not improve model evidence. Importantly, the main findings were consistent across both approaches.

Both analyses revealed that interindividual differences in LI_FFA_ were best explained by a face-specific modulation of the self-connection of the left OFA. In contrast, LI_OFA_ was associated with face-specific modulation of interregional connectivity, most prominently the interhemispheric connections between the FFAs as well as the intrahemispheric feedforward connections from the left OFA to the left FFA and from the right OFA to the right FFA. However, the latter connections did not reach the posterior probability threshold of 0.75 in the exploratory analysis. When averaging over connectivity parameters, only the LI_FFA_-related modulation of self-inhibition surpassed the posterior probability threshold of 0.75, whereas none of the LI_OFA_-related parameters did so. This pattern likely reflects a broader distribution of explanatory weight across multiple connectivity parameters for LI_OFA_ compared with LI_FFA_, suggesting a more focal mechanistic contribution for LI_FFA_ and a more distributed one for LI_OFA_.

### 4.1 Mechanistic explanations of inter-individual differences in FFA lateralization

In DCM, self-connections are components of the model’s intrinsic (within-region) connectivity. They reflect the self-regulatory dynamics of a brain region, specifically its tendency to suppress or dampen its own activity over time (Friston et al., 2003; Zeidman, Jafarian, Seghier, et al., 2019). By default, self-connections are negative, representing the natural decay of neural activity in the absence of external input. These connections can be modulated by experimental conditions. Such modulations are often interpreted as changes in neuronal gain or excitability of the respective region (e.g., Zeidman, Jafarian, Seghier, et al., 2019). A stronger (more negative) self-connection indicates greater self-inhibition, meaning the region returns to baseline more quickly. Conversely, a weaker (less negative) self-connection suggests reduced inhibition or increased excitability, potentially allowing for more persistent or amplified responses to incoming stimuli. Such modulations are, therefore, critical for understanding a region’s contribution to task-related neural processing.

In the present study, we allowed for face-specific modulations of the self-connections of the OFAs and the FFAs, while excluding the self-connections of the early visual cortex regions. Our results revealed that inter-individual differences in FFA lateralization were specifically associated with a modulation of the self-inhibition of the left OFA. Stronger left-hemispheric lateralization was associated with an attenuation of self-inhibition, indicating higher activation of this region in individuals with more left-lateralized FFA activity.

Three main conclusions emerge. First, FFA lateralization appears to be driven mainly by local, regional properties rather than by asymmetric interhemispheric or intrahemispheric connectivity. While prior work has emphasized interhemispheric interactions—such as asymmetric information transfer, interhemispheric inhibition, or differential hemispheric recruitment (Jin et al., 2024; McIntosh et al., 1994; Stephan et al., 2005, 2007; Zhu et al., 2025)—our findings suggest that individual differences are better explained by local modulations. Notably, both the winning model and the second- and third-most likely models identified in model selection contained only self-connection modulations, with no interregional connectivity changes, reinforcing the dominant role of local network properties.

Second, these lateralization differences are not equally driven by both hemispheres. One might have expected stronger leftward lateralization to result from either increased left-hemispheric activation, decreased right-hemispheric activation, or a combination of both. Our data indicate that modulations in left-hemispheric regions are the primary contributors, highlighting the prominent role of the left hemisphere in shaping lateralization and supporting the idea that hemispheric specialization within a single region can dominate lateralization effects.

Third, changes in FFA lateralization originate outside the FFA itself, at the level of the OFA. This underscores the network nature of face processing: activation differences in a given region need not be explained solely by local mechanisms (e.g., changes in self-inhibition of the left or right FFA, or direct modulation of inputs to the FFA), but can arise from modulations elsewhere in the network. Such distributed effects highlight the interactive and interconnected character of the underlying circuitry.

### 4.2 Mechanistic explanations of inter-individual differences in OFA lateralization

In contrast to the FFA, OFA lateralization was not linked to a single network parameter. Instead, inter-individual differences in LI_OFA_ were explained by multiple interregional connectivity parameters, with the most prominent being the interhemispheric connections between the left and right FFAs. Specifically, increased left-lateralization of the OFA was associated with stronger modulation right-to-left FFA input and weaker left-to-right FFA input.

These findings indicate that OFA lateralization is primarily driven by interhemispheric dynamics, consistent with the classic idea that hemispheric dominance emerges from cross-hemispheric interactions. This interpretation aligns with Porcu et al. (2024), who observed a negative correlation between intrahemispheric functional integration (“functional lateralization”) and interhemispheric connectivity.

As with the FFA, it is noteworthy that differences in OFA lateralization are not necessarily explained by changes within the OFA itself. Rather, variability in OFA lateralization appears to be primarily driven by interhemispheric couplings at other levels of the network, specifically involving the FFAs. This pattern underscores again the network-level nature of lateralization, showing that hemispheric dominance can emerge from interactions elsewhere in the system, rather than solely from local mechanisms within a given region.

### 4.3 Limitations

Finally, we would like to acknowledge a number of limitations of our study. First, the temporal resolution of fMRI is limited. The lateralization of FFA and OFA activity is unlikely to be static. Rather, it may fluctuate over time—for example, starting with a more bilateral activation pattern and subsequently evolving into a more lateralized representation (Meng et al., 2012). However, capturing such dynamic changes exceeds the temporal resolution of fMRI as used in the present study. Consequently, our findings are likely limited to characterizing the network influences on average lateralization values, rather than on their temporal dynamics.

Second, we tested neural models across a wide parameter range. Therefore, we did not expect a single model to emerge as the clear winner (i.e., with a posterior probability (Pp) > 95%). Instead of analyzing the evidence for each individual model separately, we grouped the resulting models into different families based on the three factors and analyzed the pooled evidence. It has to be noted, however, that also in these analyses only the SELF family yielded a remarkable winning model with a posterior probability of Pp > 0.95.

Third, the LI depends on several methodological choices, including the selected task, the choice of contrast (e.g., conjunction contrast vs. simple subtractive contrasts), the definition of regions of interest (e.g., large anatomical ROIs applied to all participants vs. functionally defined individual ROIs), and the activation metric used (e.g., number of activated voxels vs. t-values) (for an overview, see Jansen et al., 2006). Different approaches to LI computation may, therefore, yield different LI values, whose inter-individual variability might be driven by distinct underlying mechanisms. Consequently, the results presented in this study are, strictly speaking, specific to the particular LI computation approach employed here. It would be highly informative to systematically investigate how alternative LI definitions affect the results. However, such analyses would go beyond the scope of the current project and are planned for future work.

Fourth, in DCM the analysis results are constrained by the set of assumptions one has about the model space. We decided to have a relatively simple, yet plausible model architecture. This limits the number of regions and connections that can be included, yet ensuring stable convergence of the model estimation process. For instance, we did not incorporate the pSTS, even though it is part of the core face-processing network. However, the pSTS is primarily associated with dynamic aspects of face perception and is less central for processing static stimuli, such as those used here. Consistent with this, our localizer did not yield reliable pSTS activation. We also did not model intrahemispheric subdistinctions of the FFA (e.g., pFus-Faces vs. mFus-Faces, Chen et. al, 2023). Furthermore, we chose to not model top–down influences from FFA and OFA to EVC, as EVC served as the primary input region and early visual feedback was not the focus of our analyses. We captured possible basal visual effects by including visual modulatory self-connections on FFA and OFA regions. Finally, we restricted interhemispheric connectivity to homotopic pathways and did not include heterotopic connections between OFA and FFA across hemispheres. These cross-region interhemispheric links are typically weaker and were, therefore, considered less relevant for our model. Examining models with these type connections and their relevance for face processing remains an open question for future studies.

## 5 Conclusion

In the present study, we investigated the neural mechanisms underlying inter-individual differences in hemispheric lateralization within the face perception network, focusing on activation differences in the FFAs (LI_FFA_) and the OFAs (LI_OFA_). Our results demonstrate that lateralization in these two regions is governed by distinct network mechanisms. Inter-individual differences in FFA lateralization were best explained by a face-specific modulation of self-inhibition in the left OFA, indicating that LI_FFA_ is primarily driven by processes within the left hemisphere. In contrast, OFA lateralization was best accounted for by modulations of interregional connectivity, most prominently the interhemispheric connections between the left and right FFAs, involving contributions from both hemispheres.

Notably, and somewhat unexpectedly, changes in both FFA and OFA lateralization did not originate within the lateralized regions themselves. Instead, FFA lateralization was explained by modulations in the left OFA, whereas OFA lateralization was driven by interactions between the FFAs. Together, these findings underscore the network-based nature of face processing, demonstrating that hemispheric lateralization emerges from distributed interactions across the face perception network rather than from local changes within individual regions. Overall, our findings align with the suggestion by Blauch et al. (2025) “that local and long-range factors work synergistically to produce individual laterality patterns.” The distinction between local changes, as seen in the left OFA, and global contributions from interhemispheric interactions highlights the different mechanisms underlying hemispheric lateralization (Blauch et al., 2025). Building on our hypotheses about the mechanisms driving inter-individual differences, future studies should aim to delineate the functional specializations of the left and right homologues within the face perception network (Meng et al., 2012).

## Supplementary Material

Supplementary Material

## Data Availability

The codes are made available at the OSF website under the following link: https://doi.org/10.17605/OSF.IO/PHE9A. The DCM and PEB models can be found under following repository: https://gitlab.uni-marburg.de/stocker4/sparta.git. No source, raw or preprocessed data will be made available due to data protection laws.

## References

[IMAG.a.1219-b1] Abidi, M., Pradat, P.-F., Termoz, N., Couillandre, A., Bede, P., & de Marco, G. (2022). Motor imagery in amyotrophic lateral Sclerosis: An fMRI study of postural control. NeuroImage. Clinical, 35, 103051. 10.1016/j.nicl.2022.103051 35598461 PMC9127212

[IMAG.a.1219-b2] Ashburner, J., & Friston, K. J. (2005). Unified segmentation. NeuroImage, 26(3), 839–851. 10.1016/j.neuroimage.2005.02.018 15955494

[IMAG.a.1219-b3] Badzakova-Trajkov, G., Häberling, I. S., Roberts, R. P., & Corballis, M. C. (2010). Cerebral asymmetries: Complementary and independent processes. PLoS One, 5(3), e9682. 10.1371/journal.pone.0009682 20300635 PMC2837380

[IMAG.a.1219-b4] Bencivenga, F., Sulpizio, V., Tullo, M. G., & Galati, G. (2021). Assessing the effective connectivity of premotor areas during real vs imagined grasping: A DCM-PEB approach. NeuroImage, 230, 117806. 10.1016/j.neuroimage.2021.117806 33524574

[IMAG.a.1219-b63] Berman, M. G., Park, J., Gonzalez, R., Polk, T. A., Gehrke, A., Knaffla, S., & Jonides, J. (2010). Evaluating functional localizers: The case of the FFA. NeuroImage, 50(1), 56–71. 10.1016/j.neuroimage.2009.12.024 20025980 PMC2825676

[IMAG.a.1219-b64] Blauch, N. M., Plaut, D. C., Vin, R., & Behrmann, M. (2025). Individual variation in the functional lateralization of human ventral temporal cortex: Local competition and long-range coupling. Imaging Neuroscience, 3, imag_a_00488. 10.1162/imag_a_00488 PMC11894816 40078535

[IMAG.a.1219-b5] Bukowski, H., Dricot, L., Hanseeuw, B., & Rossion, B. (2013). Cerebral lateralization of face-sensitive areas in left-handers: Only the FFA does not get it right. Cortex, 49(9), 2583–2589. 10.1016/j.cortex.2013.05.002 23906596

[IMAG.a.1219-b6] Calzolari, S., Jalali, R., & Fernández-Espejo, D. (2023). Characterising stationary and dynamic effective connectivity changes in the motor network during and after tDCS. NeuroImage, 269, 119915. 10.1016/j.neuroimage.2023.119915 36736717

[IMAG.a.1219-b7] Catani, M., & Thiebaut de Schotten, M. (2008). A diffusion tensor imaging tractography atlas for virtual in vivo dissections. Cortex, 44(8), 1105–1132. 10.1016/j.cortex.2008.05.004 18619589

[IMAG.a.1219-b8] Chan, Y.-C., & Chou, T.-L. (2022). Effective connectivity of the amygdala during the consumption of erotic, sexual humor, and monetary rewards with a DCM-PEB approach. PLoS One, 17(12), e0279281. 10.1371/journal.pone.0279281 36580445 PMC9799303

[IMAG.a.1219-b9] Chen, C. C., Henson, R. N., Stephan, K. E., Kilner, J. M., & Friston, K. J. (2009). Forward and backward connections in the brain: A DCM study of functional asymmetries. NeuroImage, 45(2), 453–462. 10.1016/j.neuroimage.2008.12.041 19162203

[IMAG.a.1219-b10] Chen, Q., Bonduelle, S. L. B., Wu, G.-R., Vanderhasselt, M.-A., De Raedt, R., & Baeken, C. (2024). Unraveling how the adolescent brain deals with criticism using dynamic causal modeling. NeuroImage, 286, 120510. 10.1016/j.neuroimage.2024.120510 38184159

[IMAG.a.1219-b11] Chen, Z., Ji, X., Li, T., Gao, C., Li, G., Liu, S., & Zhang, Y. (2023). Lateralization difference in functional activity during Stroop tasks: a functional near-infrared spectroscopy and EEG simultaneous study. Frontiers in Psychiatry, 14, 1221381. 10.3389/fpsyt.2023.1221381 37680451 PMC10481867

[IMAG.a.1219-b12] Davies-Thompson, J., Johnston, S., Tashakkor, Y., Pancaroglu, R., & Barton, J. J. S. (2016). The relationship between visual word and face processing lateralization in the fusiform gyri: A cross-sectional study. Brain Research, 1644, 88–97. 10.1016/j.brainres.2016.05.009 27178362

[IMAG.a.1219-b13] De Winter, F.-L., Zhu, Q., Van den Stock, J., Nelissen, K., Peeters, R., de Gelder, B., Vanduffel, W., & Vandenbulcke, M. (2015). Lateralization for dynamic facial expressions in human superior temporal sulcus. NeuroImage, 106, 340–352. 10.1016/j.neuroimage.2014.11.020 25463458

[IMAG.a.1219-b14] Dietz, M. J., Zhou, Y., Veddum, L., Frith, C. D., & Bliksted, V. F. (2020). Aberrant effective connectivity is associated with positive symptoms in first-episode schizophrenia. NeuroImage. Clinical, 28, 102444. 10.1016/j.nicl.2020.102444 33039973 PMC7551359

[IMAG.a.1219-b15] Duchaine, B., & Yovel, G. (2015). A revised neural framework for face processing. Annual Review of Vision Science, 1(1), 393–416. 10.1146/annurev-vision-082114-035518 28532371

[IMAG.a.1219-b16] Esménio, S., Soares, J. M., Oliveira-Silva, P., Gonçalves, Ó. F., Friston, K., & Fernandes Coutinho, J. (2020). Changes in the effective connectivity of the social brain when making inferences about close others vs. the self. Frontiers in Human Neuroscience, 14, 151. 10.3389/fnhum.2020.00151 32410974 PMC7202326

[IMAG.a.1219-b17] Fairhall, S. L., & Ishai, A. (2007). Effective connectivity within the distributed cortical network for face perception. Cerebral Cortex (New York, N.Y.: 1991), 17(10), 2400–2406. 10.1093/cercor/bhl148 17190969

[IMAG.a.1219-b62] Fox, C. J., Iaria, G., & Barton, J. J. S. (2009). Defining the face processing network: Optimization of the functional localizer in fMRI. Human Brain Mapping, 30(5), 1637–1651. 10.1002/hbm.20630 18661501 PMC6870735

[IMAG.a.1219-b18] Frässle, S., Krach, S., Paulus, F. M., & Jansen, A. (2016). Handedness is related to neural mechanisms underlying hemispheric lateralization of face processing. Scientific Reports, 6(1), 27153. 10.1038/srep27153 27250879 PMC4890016

[IMAG.a.1219-b19] Frässle, S., Paulus, F. M., Krach, S., & Jansen, A. (2016). Test-retest reliability of effective connectivity in the face perception network. Human Brain Mapping, 37(2), 730–744. 10.1002/hbm.23061 26611397 PMC6867422

[IMAG.a.1219-b20] Frässle, S., Paulus, F. M., Krach, S., Schweinberger, S. R., Stephan, K. E., & Jansen, A. (2016). Mechanisms of hemispheric lateralization: Asymmetric interhemispheric recruitment in the face perception network. NeuroImage, 124, 977–988. 10.1016/j.neuroimage.2015.09.055 26439515

[IMAG.a.1219-b21] Friston, K. J., Harrison, L., & Penny, W. (2003). Dynamic causal modelling. NeuroImage, 19(4), 1273–1302. 10.1016/S1053-8119(03)00202-7 12948688

[IMAG.a.1219-b22] Gandolla, M., Niero, L., Molteni, F., Guanziroli, E., Ward, N. S., & Pedrocchi, A. (2021). Brain plasticity mechanisms underlying motor control reorganization: pilot longitudinal study on post-stroke subjects. Brain Sciences, 11(3), 329. 10.3390/brainsci11030329 33807679 PMC8002039

[IMAG.a.1219-b23] Haxby, J. V., Hoffman, E. A., & Gobbini, M. I. (2000). The distributed human neural system for face perception. Trends in Cognitive Sciences, 4(6), 223–233. 10.1016/S1364-6613(00)01482-0 10827445

[IMAG.a.1219-b24] He, W., Garrido, M. I., Sowman, P. F., Brock, J., & Johnson, B. W. (2015). Development of effective connectivity in the core network for face perception. Human Brain Mapping, 36(6), 2161–2173. 10.1002/hbm.22762 25704356 PMC6869188

[IMAG.a.1219-b25] He, W., & Johnson, B. W. (2018). Development of face recognition: Dynamic causal modelling of MEG data. Developmental Cognitive Neuroscience, 30, 13–22. 10.1016/j.dcn.2017.11.010 29197727 PMC6969123

[IMAG.a.1219-b26] Hofer, S., & Frahm, J. (2006). Topography of the human corpus callosum revisited—Comprehensive fiber tractography using diffusion tensor magnetic resonance imaging. NeuroImage, 32(3), 989–994. 10.1016/j.neuroimage.2006.05.044 16854598

[IMAG.a.1219-b27] Jansen, A., Menke, R., Sommer, J., Förster, A. F., Bruchmann, S., Hempleman, J., Weber, B., & Knecht, S. (2006). The assessment of hemispheric lateralization in functional MRI—Robustness and reproducibility. NeuroImage, 33(1), 204–217. 10.1016/j.neuroimage.2006.06.019 16904913

[IMAG.a.1219-b28] Jin, X., Liang, X., & Gong, G. (2024). The relationship between interhemispheric homotopic functional connectivity and left-right difference of intrahemispheric functional integration in the human brain. Imaging Neuroscience, 2, 1–18. 10.1162/imag_a_00205 PMC12272220 40800411

[IMAG.a.1219-b29] Johnstone, L. T., Karlsson, E. M., & Carey, D. P. (2020). The validity and reliability of quantifying hemispheric specialisation using fMRI: Evidence from left and right handers on three different cerebral asymmetries. Neuropsychologia, 138, 107331. 10.1016/j.neuropsychologia.2020.107331 31917204

[IMAG.a.1219-b30] Kessler, R., Rusch, K. M., Wende, K. C., Schuster, V., & Jansen, A. (2021). Revisiting the effective connectivity within the distributed cortical network for face perception. Neuroimage: Reports, 1(4), 100045. 10.1016/j.ynirp.2021.100045 40568430 PMC12172785

[IMAG.a.1219-b31] Kessler, R., Schmitt, S., Sauder, T., Stein, F., Yüksel, D., Grotegerd, D., Dannlowski, U., Hahn, T., Dempfle, A., Sommer, J., Steinsträter, O., Nenadic, I., Kircher, T., & Jansen, A. (2020). Long-term neuroanatomical consequences of childhood maltreatment: reduced amygdala inhibition by medial prefrontal cortex. Frontiers in Systems Neuroscience, 14, 28. 10.3389/fnsys.2020.00028 32581732 PMC7283497

[IMAG.a.1219-b32] Lee, S.-M., Tibon, R., Zeidman, P., Yadav, P. S., & Henson, R. (2022). Effects of face repetition on ventral visual stream connectivity using dynamic causal modelling of fMRI data. NeuroImage, 264, 119708. 10.1016/j.neuroimage.2022.119708 36280098

[IMAG.a.1219-b33] Li, J., Liu, J., Liang, J., Zhang, H., Zhao, J., Rieth, C. A., Huber, D. E., Li, W., Shi, G., Ai, L., Tian, J., & Lee, K. (2010). Effective connectivities of cortical regions for top-down face processing: A Dynamic Causal Modeling study. Brain Research, 1340, 40–51. 10.1016/j.brainres.2010.04.044 20423709 PMC3724518

[IMAG.a.1219-b34] Lohse, M., Garrido, L., Driver, J., Dolan, R. J., Duchaine, B. C., & Furl, N. (2016). Effective connectivity from early visual cortex to posterior occipitotemporal face areas supports face selectivity and predicts developmental prosopagnosia. The Journal of Neuroscience, 36(13), 3821–3828. 10.1523/JNEUROSCI.3621-15.2016 27030766 PMC4812138

[IMAG.a.1219-b35] Maldjian, J. A., Laurienti, P. J., & Burdette, J. H. (2004). Precentral gyrus discrepancy in electronic versions of the Talairach atlas. NeuroImage, 21(1), 450–455. 10.1016/j.neuroimage.2003.09.032 14741682

[IMAG.a.1219-b36] Maldjian, J. A., Laurienti, P. J., Kraft, R. A., & Burdette, J. H. (2003). An automated method for neuroanatomic and cytoarchitectonic atlas-based interrogation of fMRI data sets. NeuroImage, 19(3), 1233–1239. 10.1016/s1053-8119(03)00169-1 12880848

[IMAG.a.1219-b37] McIntosh, A. R., Grady, C. L., Ungerleider, L. G., Haxby, J. V., Rapoport, S. I., & Horwitz, B. (1994). Network analysis of cortical visual pathways mapped with PET. Journal of Neuroscience, 14(2), 655–666. 10.1523/JNEUROSCI.14-02-00655.1994 8301356 PMC6576802

[IMAG.a.1219-b38] Meng, M., Cherian, T., Singal, G., & Sinha, P. (2012). Lateralization of face processing in the human brain. Proceedings of the Royal Society B: Biological Sciences, 279(1735), 2052–2061. 10.1098/rspb.2011.1784 PMC3311882 22217726

[IMAG.a.1219-b39] Mizrachi, N., Eviatar, Z., Peleg, O., & Bitan, T. (2024). Inter- and intra-hemispheric interactions in reading ambiguous words. Cortex; a Journal Devoted to the Study of the Nervous System and Behavior, 171, 257–271. 10.1016/j.cortex.2023.09.022 38048664

[IMAG.a.1219-b40] Nagy, K., Greenlee, M. W., & Kovács, G. (2012). The lateral occipital cortex in the face perception network: An effective connectivity study. Frontiers in Psychology, 3, 141. 10.3389/fpsyg.2012.00141 22593748 PMC3349303

[IMAG.a.1219-b41] Nguyen, V. T., Breakspear, M., & Cunnington, R. (2014). Fusing concurrent EEG-fMRI with dynamic causal modeling: Application to effective connectivity during face perception. NeuroImage, 102 Pt 1, 60–70. 10.1016/j.neuroimage.2013.06.083 23850464

[IMAG.a.1219-b42] Paci, E., Lumb, B. M., Apps, R., Lawrenson, C. L., & Moran, R. J. (2023). Dynamic causal modeling reveals increased cerebellar- periaqueductal gray communication during fear extinction. Frontiers in Systems Neuroscience, 17, 1148604. 10.3389/fnsys.2023.1148604 37266394 PMC10229824

[IMAG.a.1219-b43] Penny, W. D., Stephan, K. E., Mechelli, A., & Friston, K. J. (2004). Comparing dynamic causal models. NeuroImage, 22(3), 1157–1172. 10.1016/j.neuroimage.2004.03.026 15219588

[IMAG.a.1219-b65] Pitcher, D., Dilks, D. D., Saxe, R. R., Triantafyllou, C., & Kanwisher, N. (2011). Differential selectivity for dynamic versus static information in face-selective cortical regions. NeuroImage, 56(4), 2356–2363. 10.1016/j.neuroimage.2011.03.067 21473921

[IMAG.a.1219-b66] Porcu, M., Cocco, L., Marrosu, F., Cau, R., Suri, J. S., Qi, Y., Pineda, V., Bosin, A., Malloci, G., Ruggerone, P., Puig, J., & Saba, L. (2024). Impact of corpus callosum integrity on functional interhemispheric connectivity and cognition in healthy subjects. Brain Imaging and Behavior, 18, 141–158. 10.1007/s11682-023-00814-1 37955809

[IMAG.a.1219-b44] Rho, G., Callara, A. L., Vanello, N., Gentili, C., Greco, A., & Scilingo, E. P. (2021). Odor valence modulates cortico-cortical interactions: A preliminary study using DCM for EEG. In Annual International Conference of the IEEE Engineering in Medicine and Biology Society (pp. 604–607). IEEE. 10.1109/EMBC46164.2021.9629910 34891366

[IMAG.a.1219-b45] Rossion, B., Hanseeuw, B., & Dricot, L. (2012). Defining face perception areas in the human brain: A large-scale factorial fMRI face localizer analysis. Brain and Cognition, 79(2), 138–157. 10.1016/j.neuropsychologia.2022.108279 22330606

[IMAG.a.1219-b46] Rossion, B., & Lochy, A. (2022). Is human face recognition lateralized to the right hemisphere due to neural competition with left-lateralized visual word recognition? A critical review. Brain Structure and Function, 227(2), 599–629. 10.1007/s00429-021-02370-0 34731327

[IMAG.a.1219-b47] Spedden, M. E., Beck, M. M., Christensen, M. S., Dietz, M. J., Karabanov, A. N., Geertsen, S. S., Nielsen, J. B., & Lundbye-Jensen, J. (2020). Directed connectivity between primary and premotor areas underlying ankle force control in young and older adults. NeuroImage, 218, 116982. 10.1016/j.neuroimage.2020.116982 32450250

[IMAG.a.1219-b49] Stephan, K. E., Marshall, J. C., Penny, W. D., Friston, K. J., & Fink, G. R. (2007). Interhemispheric integration of visual processing during task-driven lateralization. The Journal of Neuroscience, 27(13), 3512–3522. 10.1523/JNEUROSCI.4766-06.2007 17392467 PMC2636903

[IMAG.a.1219-b50] Stephan, K. E., Penny, W. D., Marshall, J. C., Fink, G. R., & Friston, K. J. (2005). Investigating the functional role of callosal connections with dynamic causal models. Annals of the New York Academy of Sciences, 1064(1), 16–36. 10.1196/annals.1340.008 16394145 PMC2644452

[IMAG.a.1219-b51] Stocker, J. E., Schulz, A., Thome, I., Sommer, J., Rabeneck, J., Rusch, K. M., Steinsträter, O., & Jansen, A. (2025). Interhemispheric integration in the neural face perception network: Does stimulus location matter? Imaging Neuroscience, 3, IMAG-a. 10.1162/imag.a.17 PMC12320007 40800977

[IMAG.a.1219-b52] Thome, I., García Alanis, J. C., Volk, J., Vogelbacher, C., Steinsträter, O., & Jansen, A. (2022). Let’s face it: The lateralization of the face perception network as measured with fMRI is not clearly right dominant. NeuroImage, 263, 119587. 10.1016/j.neuroimage.2022.119587 36031183

[IMAG.a.1219-b54] Wilke, M., & Lidzba, K. (2007). LI-tool: A new toolbox to assess lateralization in functional MR-data. Journal of Neuroscience Methods, 163(1), 128–136. 10.1016/j.jneumeth.2007.01.026 17386945

[IMAG.a.1219-b55] Wilke, M., & Schmithorst, V. J. (2006). A combined bootstrap/histogram analysis approach for computing a lateralization index from neuroimaging data. NeuroImage, 33(2), 522–530. 10.1016/j.neuroimage.2006.07.010 16938470

[IMAG.a.1219-b56] Yc, C., Cy, W., & Tl, C. (2023). Money or funny: Effective connectivity during service recovery with a DCM-PEB approach. Biological Psychology, 176, 108464. 10.1016/j.biopsycho.2022.108464 36435295

[IMAG.a.1219-b57] Zeidman, P., Friston, K., & Parr, T. (2023). A primer on Variational Laplace (VL). NeuroImage, 279, 120310. 10.1016/j.neuroimage.2023.120310 37544417 PMC10951963

[IMAG.a.1219-b58] Zeidman, P., Jafarian, A., Corbin, N., Seghier, M. L., Razi, A., Price, C. J., & Friston, K. J. (2019). A guide to group effective connectivity analysis, part 1: First level analysis with DCM for fMRI. NeuroImage, 200, 174–190. 10.1016/j.neuroimage.2019.06.031 31226497 PMC6711459

[IMAG.a.1219-b59] Zeidman, P., Jafarian, A., Seghier, M. L., Litvak, V., Cagnan, H., Price, C. J., & Friston, K. J. (2019). A guide to group effective connectivity analysis, part 2: Second level analysis with PEB. NeuroImage, 200, 12–25. 10.1016/j.neuroimage.2019.06.032 31226492 PMC6711451

[IMAG.a.1219-b60] Zhu, M., Wang, X., Zhao, X., & Cai, Q. (2025). Intrahemispheric white matter asymmetries and interhemispheric connections underlying the lateralization of language production and spatial attention in left-handers. Neurobiology of Language, 6, nol_a_00153. 10.1162/nol_a_00153 PMC11740161 39830069

[IMAG.a.1219-b61] Zhuang, Q., Qiao, L., Xu, L., Yao, S., Chen, S., Zheng, X., Li, J., Fu, M., Li, K., Vatansever, D., Ferraro, S., Kendrick, K. M., & Becker, B. (2023). The right inferior frontal gyrus as pivotal node and effective regulator of the basal ganglia-thalamocortical response inhibition circuit. Psychoradiology, 3, kkad016. 10.1093/psyrad/kkad016 PMC10917375 38666118

