# Supplementary material for: Causal mechanisms of individual differences in hemispheric lateralization of the face perception network: A DCM-PEB approach
Source: Imaging Neurosci (Camb). 2026 Apr 24;4:IMAG.a.1219. doi: 10.1162/IMAG.a.1219 (PMC13112208; doi:10.1162/IMAG.a.1219)
Supplement: Supplementary Material [file IMAG.a.1219_supp.pdf]

Supplementary Material for the study on "Causal Mechanisms of Individual Differences in Hemispheric Lateralization of the Face Perception Network: A DCM-PEB approach"

Julia Elina Stocker [1], Ina Thome [1], Peter Zeidman[5], Kristin Marie Rusch [1], Jens Sommer [1], Olaf Steinsträter [1], Andreas Jansen [1]

This supplement contains additional reports on the data from the face network lateralization study. The previous study material was published under OSF https://osf.io/s8gwd/, there the pre-processed data can be retrieved. The codes used for this analysis are published at a different repository at GitHub (https://gitlab.uni-marburg.de/stocker4/sparta.git). For further information please contact the corresponding author:

Julia Elina Stocker, Department of Psychiatry and Psychotherapy, Philipps University of Marburg, Germany

Rudolf-Bultmann-Straße 8, D-35039 Marburg

E-Mail: elina.stocker@uni-marburg.de

1. Variation of the Lateralization Indices

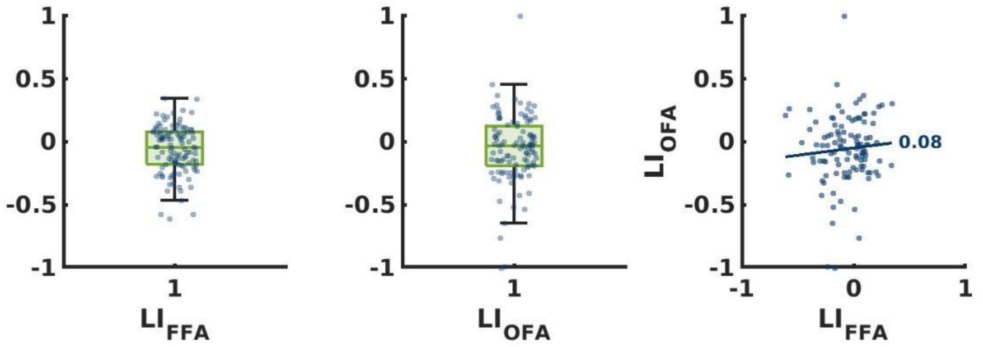

Fig S1. Lateralization measured by the Lateralization Index (LI) of FFA and OFA across 110 participants. Left. The distribution of the LI for the FFA shown in a box plot. Middle. The distribution of the LI for the OFA shown in a box plot. Right. The correlation between the LI for OFA and FFA presented with a value of 0.08.

2. ROI Group Peak Coordinates

Region of interest	x	y	z
Left OFA	-42	-86	-10
Right OFA	46	-80	-8
Left FFA	-42	-50	-20
Right FFA	42	-46	-18
Left EVC	-12	-94	-6
Right EVC	18	-86	0

Tbl T1. Group peak coordinates for conjunction contrast (faces > houses AND faces > scrambled), $p < 0.05$ FWE corrected.

3. Bayesian Parameter Averaging

Parameter Averages over 110 DCMs

Pram_cat	Param_conn	A-Matrix	B-Matrix (Faces)
self	lFFA-lFFA	0.19	1.98
inter	rFFA-lFFA	0.24	0.67
intra	lOFA-lFFA	0.10	1.22
inter	lFFA-rFFA	0.24	0.38
self	rFFA-rFFA	-0.59	-3.10
intra	rOFA-rFFA	-0.09	1.25
intra	lFFA-lOFA	0.01	-0.23
self	lOFA-lOFA	-0.90	-1.46
inter	rOFA-lOFA	0.01	-0.34
intra	rFFA-rOFA	-0.21	-0.52
inter	lOFA-rOFA	-0.26	-0.49
self	rOFA-rOFA	-0.44	1.85
input	lFFA-lEVC	0.27	-0.13
input	lOFA-lEVC	0.20	-0.43
input	lEVC-lEVC	0.62	-
input	rFFA-rEVC	0.03	-0.21
self	rOFA-rEVC	0.02	0.17
self	rEVC-rEVC	0.42	-

Tbl T2. Bayesian Parameter Averages over all 100 models (one per subject) within the GCM. Only the structural (A-Matrix) and face-modulated (B-Matrix) connectivity parameter strength are presented.

Visualization of Model Averages

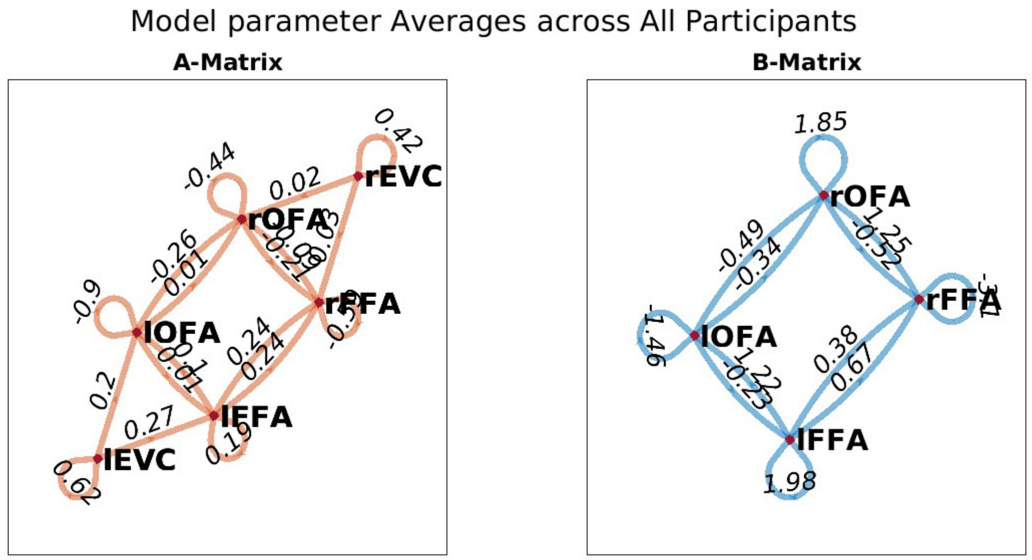

Fig S2. Face processing Network for structural (A-Matrix) and face-modulated (B-Matrix) connectivity changes. Bayesian Parameter Averages over all 100 models (one per subject) within the GCM are presented as weights on the graph edges.

4. Group-level Modulatory (B-Matrix) Parameters

PEB Model Parameters

Param_id	Pram_cat	Param_conn	Mean	LI-FFA	LI-OFA	Handedness	Gender	Age
B(1.1.1)	self	lFFA-lFFA	-0.83	1.15	-0.04	0.05	-0.29	-0.11
B(2.1.1)	inter	lFFA-rFFA	0.57	1.25	-1.19	-0.24	0.16	-0.09
B(3.1.1)	intra	lFFA-lOFA	1.00	0.55	-0.27	-0.10	-0.19	-0.12
B(1.2.1)	inter	rFFA-lFFA	-0.01	-0.63	1.33	-0.16	-0.08	0.11
B(2.2.1)	self	rFFA-rFFA	-0.32	0.02	-0.52	-0.16	0.86	0.37
B(4.2.1)	intra	rFFA-rOFA	0.62	-0.47	1.24	-0.02	-0.16	-0.10
B(1.3.1)	intra	lOFA-lFFA	-0.38	1.11	-0.82	0.12	-0.19	-0.10
B(3.3.1)	self	lOFA-lOFA	0	-2.60	-0.65	0.23	0.36	-0.09
B(4.3.1)	inter	lOFA-rOFA	-0.43	0.58	-0.50	0.06	-0.05	0.28
B(2.4.1)	intra	rOFA-rFFA	-0.42	-0.35	0.39	0.02	0.08	0.18
B(3.4.1)	inter	rOFA-lOFA	-0.33	-1.32	0.37	0.02	0.09	0.20
B(4.4.1)	self	rOFA-rOFA	-0.18	1.15	0.49	-0.19	-0.03	-0.03
B(1.5.1)	input	lEVC-lFFA	0.41	0.09	-0.48	-0.01	0.18	-0.10
B(3.5.1)	input	lEVC-lOFA	0.05	-0.11	-0.31	0.07	0.15	-0.21
B(2.6.1)	input	rEVC-rFFA	0.33	-0.66	-0.37	0.04	-0.07	-0.10
B(4.6.1)	input	rEVC-rOFA	0.13	-0.20	-0.31	0.07	0.14	0.10
B(1.1.2)	self	lFFA-lFFA	-0.10	-0.19	0.20	-0.07	0.02	0.47
B(2.2.2)	self	rFFA-rFFA	0.35	-0.04	-0.59	0.16	-0.07	0.06
B(3.3.2)	self	lOFA-lOFA	0.36	0.87	0.38	-0.24	-0.37	0.27
B(4.4.2)	self	rOFA-rOFA	0.01	-1.56	-0.13	0.14	-0.37	0.26
B(5.5.2)	self	lEVC-lEVC	-0.14	-0.23	0	-0.04	-0.02	0.03
B(6.6.2)	self	rEVC-rEVC	-0.27	0.11	-0.19	0.03	-0.07	-0.03

Tbl T3. General PEB model parameters of the modulatory effects of faces (rows 1-16) or visual input (17-22).

Family-wise B-Parameters after BMA (all Families)

Param_id	Pram_cat	Param_conn	Mean	LI-FFA	LI-OFA	Handedness	Gender	Age
B(1.1.1)	self	lFFA-lFFA	**-0.85**	0.21	-0.04	0.08	-0.21	-0.11
B(2.1.1)	inter	lFFA-rFFA	**0.57**	0	-1.17	-0.20	0.25	-0.13
B(3.1.1)	intra	lFFA-lOFA	**1.00**	0.01	-0.19	-0.09	-0.16	-0.14
B(1.2.1)	inter	rFFA-lFFA	**-0.02**	0	1.31	-0.18	-0.15	0.13
B(2.2.1)	self	rFFA-rFFA	**-0.26**	0	-0.53	-0.16	0.86	0.36
B(4.2.1)	intra	rFFA-rOFA	**0.62**	0	1.25	-0.03	-0.20	-0.10
B(1.3.1)	intra	lOFA-lFFA	**-0.37**	0	-0.82	0.17	-0.08	-0.12
B(3.3.1)	self	lOFA-lOFA	**0.04**	**-2.27**	-0.60	0.21	0.30	-0.07
B(4.3.1)	inter	lOFA-rOFA	**-0.42**	0	-0.53	0.09	0.02	0.27
B(2.4.1)	intra	rOFA-rFFA	**-0.43**	0	0.38	0.02	0.05	0.18
B(3.4.1)	inter	rOFA-lOFA	**-0.33**	-0.02	0.32	-0.02	0.01	0.22
B(4.4.1)	self	rOFA-rOFA	**-0.17**	0.25	0.50	-0.16	0	-0.04
B(1.5.1)	input	lEVC-lFFA	**0.41**	0	-0.48	-0.01	0.15	-0.10
B(3.5.1)	input	lEVC-lOFA	**0.05**	0	-0.31	0.06	0.13	-0.22
B(2.6.1)	input	rEVC-rFFA	**0.34**	0	-0.39	0.02	-0.11	-0.09
B(4.6.1)	input	rEVC-rOFA	**0.14**	0	-0.30	0.06	0.10	0.13
B(1.1.2)	self	lFFA-lFFA	0	0	0.19	-0.07	0	0.48
B(2.2.2)	self	rFFA-rFFA	0	0	-0.61	0.16	-0.06	0.05
B(3.3.2)	self	lOFA-lOFA	0	0	0.42	-0.21	-0.31	0.24
B(4.4.2)	self	rOFA-rOFA	0	0	-0.14	0.09	-0.49	0.28
B(5.5.2)	self	lEVC-lEVC	-0.13	-0.21	0	-0.05	-0.02	0.03
B(6.6.2)	self	rEVC-rEVC	-0.27	0.12	-0.19	0.03	-0.07	-0.03

Tbl T4. PEB model parameters of the family-analysis. This shows the averages over all models. weighted by their family probabilities. Bold values are above 0.75 free energy threshold.

Random Reduced B-Parameters (BMR-Analysis)

Param_id	Pram_cat	Param_conn	Mean	LI-FFA	LI-OFA	Handedness	Gender	Age
B(1.1.1)	self	lFFA-lFFA	**-0.82**	0	0	0	0	0
B(2.1.1)	inter	lFFA-rFFA	**0.57**	1.02	**-1.16**	-0.12	0	0
B(3.1.1)	intra	lFFA-lOFA	**1.02**	0	0	0	0	0
B(1.2.1)	inter	rFFA-lFFA	0	0	**1.01**	0	0	0
B(2.2.1)	self	rFFA-rFFA	**-0.37**	0	0	0	0	0
B(4.2.1)	intra	rFFA-rOFA	**0.65**	0	0.45	0	0	0
B(1.3.1)	intra	lOFA-lFFA	**-0.37**	0	-0.36	0	0	0
B(3.3.1)	self	lOFA-lOFA	0	**-1.75**	0	0	0	0
B(4.3.1)	inter	lOFA-rOFA	**-0.40**	0	0	0	0	0
B(2.4.1)	intra	rOFA-rFFA	**-0.44**	0	0	0	0	0
B(3.4.1)	inter	rOFA-lOFA	**-0.32**	-0.71	0	0	0	0
B(4.4.1)	self	rOFA-rOFA	0	0	0	0	0	0
B(1.5.1)	input	lEVC-lFFA	**0.41**	0	0	0	0	0
B(3.5.1)	input	lEVC-lOFA	0	0	0	0	0	0
B(2.6.1)	input	rEVC-rFFA	**0.33**	-0.48	0	0	0	0
B(4.6.1)	input	rEVC-rOFA	0	0	0	0	0	0
B(1.1.2)	self	lFFA-lFFA	0	0	0	0	0	0.29
B(2.2.2)	self	rFFA-rFFA	**0.35**	0	0	0	0	0
B(3.3.2)	self	lOFA-lOFA	**0.36**	0	0	0	0	0
B(4.4.2)	self	rOFA-rOFA	0	-0.72	0	0	0	0

B(5.5.2)	self	lEVC-lEVC	**-0.14**	0	0	0	0	0
B(6.6.2)	self	rEVC-rEVC	**-0.27**	0	0	0	0	0

Tbl T5. PEB model parameters of the BMR-analysis. This shows the averages over all models. weighted by their probabilities. Bold values are above 0.75 free energy threshold.

5. Extended Analysis

Only EVC Model B-Parameters (Family-Analysis)

In order to check the robustness of our analysis, we also reran the analysis with a different model, containing no visual modulations on the OFA and FFA self-connections. Thus only EVC regions could be modulated by visual input.

Param_id	Pram_cat	Param_conn	Mean	LI-FFA	LI-OFA
B(1.1.1)	self	lFFA-lFFA	-1.07	0.14	-0.27
B(2.1.1)	inter	lFFA-rFFA	0.83	0.07	-0.81
B(3.1.1)	intra	lFFA-lOFA	1.19	0	0.62
B(1.2.1)	inter	rFFA-lFFA	0.01	-0.35	0.99
B(2.2.1)	self	rFFA-rFFA	0.12	0.02	0
B(4.2.1)	intra	rFFA-rOFA	0.97	-0.07	1.07
B(1.3.1)	intra	lOFA-lFFA	-0.40	0.36	-0.95
B(3.3.1)	self	lOFA-lOFA	0.16	-0.38	0.16
B(4.3.1)	inter	lOFA-rOFA	-0.42	0.09	-1.22
B(2.4.1)	intra	rOFA-rFFA	-0.52	-0.09	0.61
B(3.4.1)	inter	rOFA-lOFA	-0.47	-0.03	0.19
B(4.4.1)	self	rOFA-rOFA	0.17	0.01	-0.74
B(1.5.1)	input	lEVC-lFFA	0.41	0	-0.19
B(3.5.1)	input	lEVC-lOFA	-0.15	0	-0.94
B(2.6.1)	input	rEVC-rFFA	0.37	0	-0.45
B(4.6.1)	input	rEVC-rOFA	0.02	-0.05	-0.23
B(5.5.2)	self	lEVC-lEVC	-0.11	0.1	-0.06
B(6.6.2)	self	rEVC-rEVC	-0.29	0.22	-0.08

Tbl T6. PEB model parameters of the extended analysis (onlyEVC visual modulation). This shows the averages over all models. weighted by their probabilities.
